# A modified trajectory of kyphoplasty *via* superior pedicle notch for osteoporotic vertebral compression fractures: Technique note and clinical result

**DOI:** 10.3389/fsurg.2022.1012160

**Published:** 2022-09-15

**Authors:** Yi Jiang, Jian Li, Shuai Yuan, Rujun Zuo, Chang Liu, Jiexun Zhang, Ming Ma

**Affiliations:** Department of Orthopedics (Minimally Invasive Spine Surgery Branch), Beijing Haidian Hospital (Haidian Section of Peking University Third Hospital), Beijing, China

**Keywords:** kyphoplasty, lumbar, extra-pedicular, vertebral body, trajectory

## Abstract

**Background:**

Percutaneous extra-pedicular kyphoplasty can achieve better clinical results than transpedicular kyphoplasty. However, lumbar segment artery injury as a disaster complication limits its clinical application.

**Objective:**

To describe and evaluate a modified trajectory of kyphoplasty for the treatment of osteoporotic vertebral compression fractures (OVCF).

**Methods:**

Eighty-one patients who underwent percutaneous kyphoplasty (PKP) for lumbar OVCF at our hospital between May 2017 and May 2021 were enrolled. The patients were divided into an observation group (*via* the superior pedicle approach) and a control group (*via* the transpedicular approach) according to the surgical trajectory. The surgical procedure was described in detail, and the imaging parameters were recorded. Preoperative and postoperative clinical data were collected for statistical analysis.

**Results:**

PKP *via* the superior pedicle notch approach could offer large abduction and cranial inclination angles without serious complications. The rate of paravertebral leakage was significantly lower in the observation group than in the control group. Surgery with a superior pedicle notch approach had a shorter operative time and fewer fluoroscopies.

**Conclusions:**

PKP *via* the superior pedicle notch approach is a modified extra-pedicular approach for lumbar osteoporotic vertebral compression fractures. This trajectory is an easy-to-use target position because it enters the vertebral body directly. A shorter operative time and lower radiation exposure can enhance recovery after surgery.

## Introduction

Percutaneous kyphoplasty (PKP) is a procedure based on percutaneous vertebroplasty (PVP) that uses an expandable balloon to provide good resetting of the compressed vertebral body followed by cement augmentation (1). The transpedicular approach is the most commonly used surgical approach for PKP and is divided into unilateral and bilateral punctures. Some scholars (2–4) have reported that a unilateral approach to PKP can achieve similar clinical results to the bilateral approach, while reducing operative time, radiation exposure, and the incidence of surgical complications. However, owing to the anatomical characteristics of the pedicle, unilateral puncture does not easily reach the center of the vertebral body, and there is a risk of asymmetric distribution of bone cement, which subsequently results in vertebral biomechanical imbalance and even vertebral refracture (5). To compensate for this deficiency, it has been suggested that a parallel extra-pedicular approach be used, which has a lower probability of puncture failure and allows bilateral cement dispersion (6, 7). However, Liu et al. (8) showed that the lumbar segmental artery is closely related to the trajectory of this approach, and that puncture is prone to segmental artery injury. Heo (9) reported a case of severe segmental artery injury resulting in hemorrhagic shock due to this approach. Few studies (10, 11) tried to modify the extra-pedicular approach through cadaveric and clinical studies to avoid this disaster. There are few cases to compare these approaches as well as a lack of accurate technical descriptions and analyses. Therefore, further studies on the puncture path, surgical details, and clinical efficacy of the extra-pedicular approach to PKP are necessary. In this study, the superior pedicle notch was used as the bony entry point for the modified extra-pedicular approach in our center, which was applied in osteoporotic vertebral compression fracture (OVCF) of the lumbar spine and compared with the bilateral transpedicular approach to PKP to evaluate its advantages and disadvantages.

## Materials and methods

### Patient population

A retrospective collection of patients who underwent PKP for lumbar OVCF at our hospital from May 2017 to May 2021 was enrolled in this study. The inclusion criteria were as follows: low energy injury resulting in fracture and pain; area of pain consistent with imaging without neurological symptoms; bone density *T*-value <−2.5; and preoperative imaging data confirming the diagnosis of fresh compression fracture with AO classification of A1 fracture. The exclusion criteria were as follows: combination of metabolic bone disease; vertebral hemangioma; osteolytic vertebral metastases; vertebral burst fracture; posterior ligament complex injury; Kümmell's disease; and incomplete imaging data. A total of 81 patients were enrolled. Among them, 74 patients had single vertebral fractures and seven had double vertebral fractures, totaling 88 vertebrae. Forty-seven vertebrae underwent the unilateral superior pedicle notch approach (observation group), and 41 vertebrae underwent the bilateral transpedicular approach (control group). Data on age, sex, body mass index (BMI), fracture segment, preoperative visual analog scale (VAS) score, and preoperative Oswestry Disability Index (ODI) scores were collected from the patients in the enrolled group. The measurement data are expressed as (±SD). Count data were expressed as percentages. Table 1 presents the general statistics.

**Table 1:** General information and comparison of patients enrolled in the group.

	Age	BMI	Gender		VAS	ODI
			Male	Female		
Observation group	77.6 ± 8.0	23.80 ± 2.89	11	36	7.91 ± 1.02	86.34 ± 4.63
Control group	76.9 ± 9.6	22.97 ± 2.39	12	29	8.22 ± 0.69	84.63 ± 4.37
χ^2^ value			0.390			
*T*-value	0.396	1.455			−1.661	1.771
*P*-value	0.693	0.149	0.532	0.101	0.080	

### Design of the ideal trajectory

**Figure 1:**
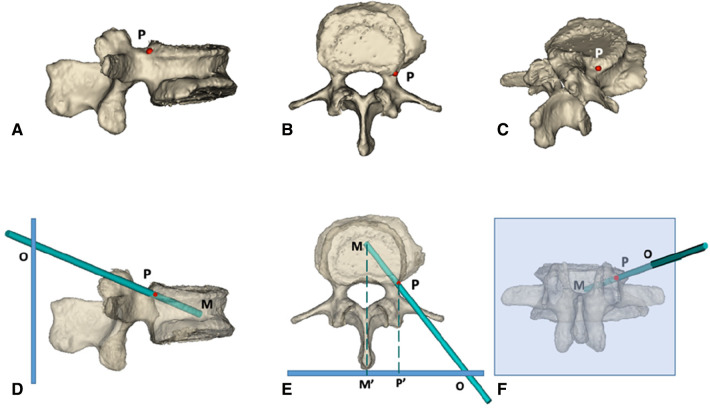
3d simulation diagram of PKP *via* superior pedicle notch approach. (**A–C**) the side view, top view and posterior view of the bony puncture entry point of PKP in the ideal state, respectively; the puncture needle is simulated with a long green rod, and the red *P* point is the bony entry point. (**D–F**) the models of the vertebral body after transparent treatment; the thick blue line in (**D,E**) and the light blue box in (**F**) represent the skin, and the green dotted line represents the vertebral body midline Point M is the final puncture target point, located in the midline of the orthotropic vertebral body and the anterior middle third of the lateral vertebral body; point *P* is the bony entry point, located at the superior pedicle notch; point O: is the skin entry point, derived from the extension of the line connecting point M and point *P* intersecting with the skin. M′ and P′ points are the projection points of M and P points on the skin, respectively.

**Figure 2:**
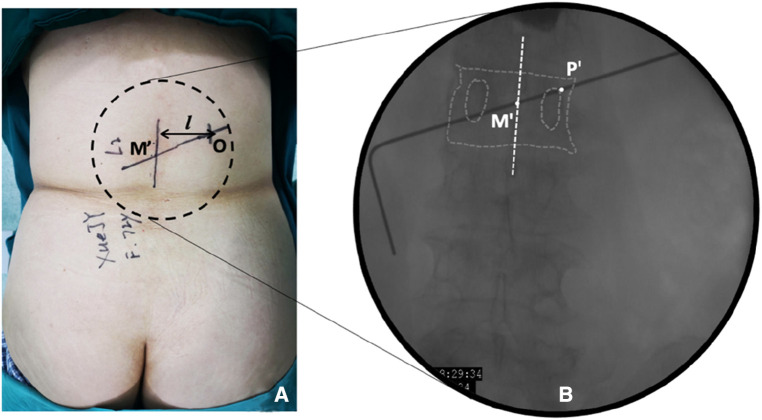
Establishment of body surface marker lines in the observation group. (**A**) Setting the dorsal skin marker line. Point M′ is the projection point of the final puncture target in the skin, and point O is the skin entry point; (**B**) Schematic diagram of the orthogonal vertebral body projection. Point M′ is the central projection point of the vertebral body, and point P′ is the bony approach projection point.

The DICOM format file of the CT scan was retrieved, the fractured vertebral body was modeled, and the puncture path was simulated using MIMICS software 17.0(Materialise Interactive Medical Image Control System, Materialise Company, Leuven, Belgium) (Figure 1). The bony entry point (red point *P*, Figures 1A–C) was set in the junction of the superior pedicle notch and vertebrae; the destination (point M, Figures 2D–F) was in the midline of the vertebral body in the cross-section and the anterior middle third of the lateral vertebral body; point *P* was the bony entry point. The line between the final target point and the bony entry point is the ideal trajectory (line MP, Figures 2D–F), and the intersection of its extension with the skin is the skin entry point (point O, Figures 2D–F).

### Surgery procedure

Control group: The specific operation was performed according to expert consensus on the standardized operation of PKP, 2018 version (11).

**Figure 3:**
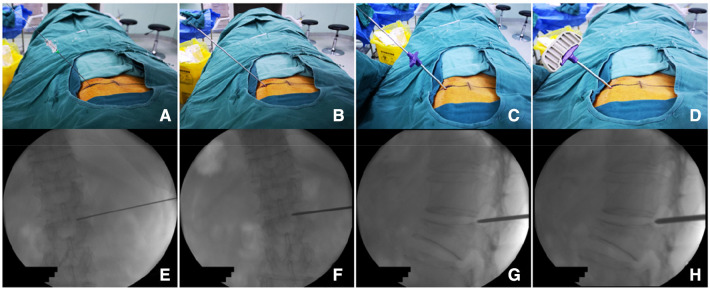
Procedure of percutaneous puncture to the bony entry point. (**A**) puncture needle puncture positioning to *P* point and local anesthetic injection; (**B**) puncture sleeve replacing the puncture needle; (**C**) working sleeve replacing the puncture sleeve; (**D**) after anchoring the bony entry point, replacing it with a Jamshidi needle to further enter into the vertebral body. (**E–H**) Represents the situation monitored by x-ray during operation.

**Figure 4:**
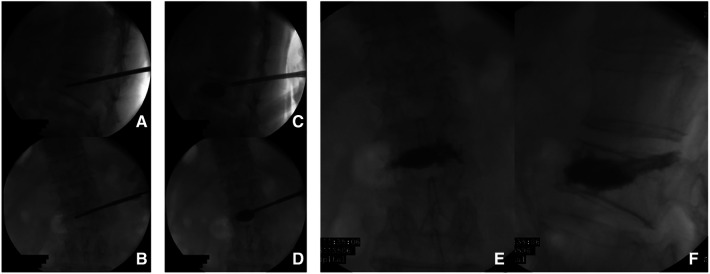
Percutaneous kyphoplasty using a single-balloon device preformed in L2 lumbar vertebra. (**A–D**) the imaging of cement augmentation after kyphoplasty (**D,E**).

Observation group: The patient was placed in the prone position. The centroid of the vertebral body (point M′, Figures 2A,B) and the outer superior edge of the pedicle projection (point P′, Figure 2B) were marked using fluoroscopy, and the marker lines from M′ and P′ were drawn on the skin (Figure 2A, line M′O). The appropriate distance L can be measured on preoperative CT (Figure 2A, point O). All patients were under local anesthesia, including the skin, subcutaneous tissue, articular synovial joint, and local periosteum at the bony entry point. A 18G puncture needle (Figure 3A) was inserted from the skin puncture point with 40° of abduction following the direction of the marker line (6), and the needle tip was slipped over the supra-articular process, then the needle tip reached the junction of the superior pedicle notch and the vertebrae. Under anterior-posterior (Figure 3A) and lateral fluoroscopy views, the tip of the needle is in an ideal position (Figures 3A,G), which is the bony entry point. The needle core was removed, and the sequence of the guide wire-dilating cannula-hollow puncture sleeve was gradually expanded to establish a puncture channel (Figure 4). For stability, the Jamshidi needle was replaced and continued to enter 1 cm by tapping. The expansion of the bone channel, balloon expansion, bone cement injection (Figure 4), and remaining steps were the same as those in the control group. Communication with the patient was maintained at all times during puncture and augmentation, and the puncture route was promptly corrected if lower-limb neuralgia occurred.

### Outcome measurements

#### Imaging-related observation index

Vertebral body height and its improvement rate: x-rays were taken before surgery and 2 days after surgery, with the injured vertebra as the center, and the anterior margin and middle height of the lateral vertebral body were measured using miPlatform 3.0 software (Haina Medical Information Beijing Software Technology Co., Ltd.). The vertebral height improvement rate was evaluated according to the method of Yi et al. (12): vertebral body height (mm) = (anterior margin height + middle height)/2, expected injured vertebral height (mm) = (upper adjacent vertebral body height + lower adjacent vertebral body height)/2, and injured vertebral height improvement rate = (postoperative injured vertebral height–preoperative injured vertebral height)/(injured vertebral height expectation–preoperative injured vertebral height) × 100%.

Bone cement distribution and leakage: CT was re-examined 2 days after surgery, and the distribution of bone cement (unilateral distribution: bone cement is distributed only on one side of the vertebral body midline, bilateral distribution. It is distributed on both sides of the vertebral body midline), and leakage of bone cement (recorded by leakage site) was observed using miPlatform 3.0.

**Figure 5:**
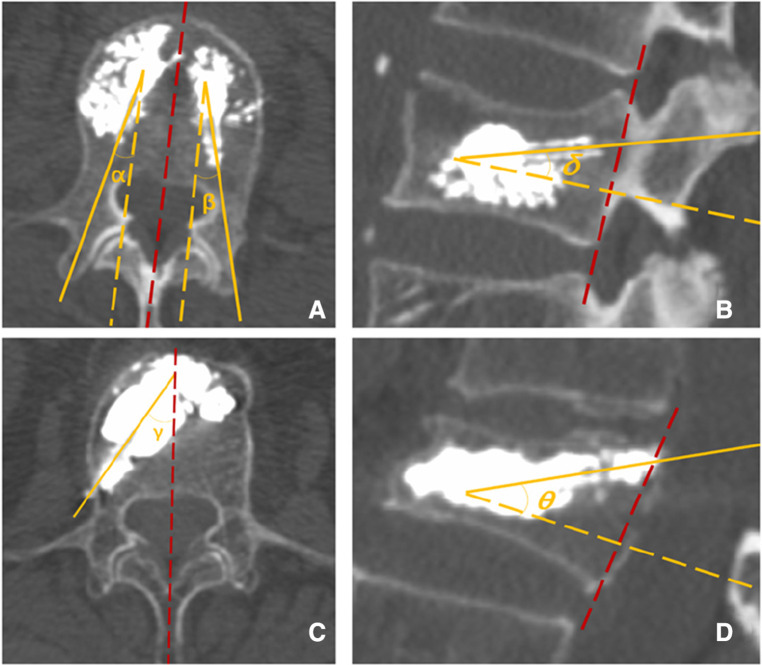
(**A**) The abduction angle *α* and *β via* the transpedicular approach. (**B**) The cranial inclination angle *δ via* transpedicular approach. (**C**) The abduction angle *γ via* the superior pedicle notch approach. (**D**) The cranial inclination angle *θ via* the t superior pedicle notch.

Puncture angle: On the postoperative 3D reconstructed CT, the abduction angle (the angle was set between the puncture trajectory and the median line on the section where the puncture needle was located. The average value of both sides was taken for the control group (Figure 5A,C), and the cranial inclination angle was set between the angle between the projection of the puncture trajectory on the sagittal plane of the vertebral body and the vertical line of the posterior edge of the vertebral body. The average value of both sides was taken for the control group (Figure 5B,D), and both were measured according to the puncture needle tracked.

#### Surgical operation-related observation index

We recorded the puncture fluoroscopy time, cement-enhanced fluoroscopy time, procedure time, and volume of cement injection. The VAS scores were obtained on postoperative day 2. The ODI scores were recorded at the last follow-up.

### Statistical analysis

SPSS 18.0 software (Statistical Product Service Solutions, IBM Corporation, New York, America) was applied for statistical analysis. The count data were compared using the chi-square test, and the measurement data were compared using an independent sample *t*-test.

## Results

All 74 patients enrolled in the group successfully completed the surgery without intraoperative puncture nerve root injury, spinal cord injury, segmental artery injury, hemorrhage, device fracture, or balloon rupture. One patient in the observation group experienced nerve root harassment during puncture, and after adjusting the puncture direction and applying gradual sleeve expansion, no further nerve irritation occurred. In the control group, one patient developed an intraoperative bone cement reaction, and the patient's vital signs recovered smoothly after ceasing the injection immediately and administering methylprednisolone 500 mg intravenously. The follow-up period for all patients was over one year.

### Imaging results

There was no statistically significant difference in the rate of improvement of vertebral body height between the two groups (*P* > 0.05). The abduction and cranial inclination angles in the observation group were significantly greater than those in the control group (*P* < 0.05) (Table 2). There was no significant difference in the unilateral distribution rate of bone cement between the two groups (*P* > 0.05). All the cement leakages in this study were asymptomatic. The rate of paravertebral leakage in the observation group was significantly lower than that in the control group (*P* < 0.05). There were no significant differences in the intradiscal, intervertebral disc, and intervertebral foramen leakage rates between the two groups (*P* > 0.05) (Table 3).

**Table 2:** Rate of improvement in vertebral body height, abduction angle, and cranial inclination angle (*x* ± s).

	Vertebral body height improvement rate (%)	Abduction angle (°)	Cranial Inclination angle (°)
Observation group	47.11 ± 21.16	30.15 ± 4.72	28.22 ± 3.08
Control group	48.70 ± 22.10	13.95 ± 3.62	14.90 ± 3.33
*T*-value	−0.344	17.862	17.510
*P*-value	0.731	0.000*	0.000*

*Showed significant differences between the two groups (*P* < 0.05).

**Table 3:** Distribution and leakage of bone cement (%).

	Observation group	Control group	χ^2^ values	*P*-value
Unilateral distribution of bone cement	3 (6.8%)	1 (2.4%)	0.785	0.376
Paravertebral leakage	12 (25.5%)	21 (51.2%)	6.165	0.013*
Intralesional leakage	12 (25.5%)	4 (9.8%)	3.663	0.056
Intervertebral Disc Leakage	6 (14.3%)	2 (4.9%)	1.649	0.199
Extraforaminal leakage	1 (2.1%)	0	0.882	0.438

*Showed significant differences between the two groups (*P* < 0.05).

### Surgical operation-related indicators

The observation group had fewer fluoroscopies during surgery than the control group, which was statistically different (*P* < 0.05). The observation group had a shorter operative time, which was significantly different from that of the control group (*P* < 0.05). There was no statistically significant difference in the amount of bone cement injected between the two groups (*P* > 0.05).

The postoperative VAS scores in the observation and control groups were 2.01 ± 0.77 and 1.93 ± 0.56 points, respectively. There were no statistically significant differences between the two groups (*P* > 0.05). The OID score at the last follow-up was 24.15 ± 13.10 in the observation group and 27.50 ± 10.22 in the control group, with no statistical difference.

## Discussion

PKP, a mature technique for vertebral augmentation, is commonly performed in medical institutions worldwide (13). Traditional PKP *via* the bilateral pedicular approach can achieve good vertebral height restoration and bilateral diffusion of bone cement but may result in complications during puncture, such as nerve root and spinal cord injury, arch fracture, segmental artery injury, and uneven distribution of bone cement (5, 14). In particular, for patients with pedicle dysplasia, poor visualization due to osteoporosis and scoliosis makes transpedicular puncture more difficult, so the possibility of discovering alternative puncture routes has attracted the interest of many scholars.

In 1990, Brugieres (15) first used an extra-pedicular approach for biopsy of the central thoracic vertebral body, believing that this approach would allow easier access to the vertebral body center. Later, some authors (16, 17) used this approach in PKP for OVCF of the thoracic spine and concluded that the unilateral extra-pedicular approach not only allowed cement dispersion in the center of the vertebral body but also prevented pedicle fractures and spinal cord injuries due to puncture. Ringer et al. (18) systematically reviewed different surgical approaches for vertebral augmentation and suggested that the extra-pedicular approach could also be used for the lumbar spine. They described an extra-pedicular approach *via* the lateral border of the transverse process to the vertebral body, which resulted in the puncture reaching the center of the vertebral body or even the contralateral side, allowing bilateral diffusion of the cement. However, some researchers have found that this approach is often close to segmental arteries, which poses a risk of injury and may lead to retroperitoneal hematoma or even hemorrhagic shock (9). To avoid these serious complications, Liu et al. (8) investigated the relationship between the position of the segmental artery and vertebral pedicle and showed that the segmental artery is easily damaged during the traditional extra-pedicular approach, while the lateral area of the posterior superior margin of the vertebral body is relatively safe. Cho (10) concluded that the connection between the vertebral body and superior lateral pedicle is a safe bony entry point, but the skin entry point setting and puncture trajectory were not described and were not further extended to clinical application.

In this study, we adopted the junction of the superior pedicle notch and vertebra as the bony entry point, which is stable and safe in anatomy. This approach has several advantages compared to the transpedicular approach; moreover, this trajectory contributed to the large angle of abduction and cranial inclination toward the transpedicular approach. In the observation group, the average abduction angle was 30.15 ± 4.72°, and the cranial inclination angle was 28.22 ± 3.08°. An oblique trajectory due to cranial inclination can avoid artery injury. A large abduction angle leads to the middle part of the vertebra. Before entering the vertebral body, the needle is easily repositioned because there is no bony limitation, and the entry point in front of the spinal canal effectively decreases the potential risk of spinal cord injury. However, this trajectory seems to cause irritation of the exiting nerve even after injury. To avoid this disadvantage, we performed the procedure under local anesthesia. Additionally, patient feedback played a role in safety. In order to decrease the surgical trauma and nerve injury risk, we prefer to use an 18G puncture needle instead of the Jamshidi needle.

The results of this study showed that the observation group had a shorter operative time and less radiological exposure than the control group (Table 4). The first bony entry point of the PKP *via* the superior pedicle notch is the vertebral cortex, which can enter the vertebral body directly without the manipulation of pedicle puncture, thus reducing radiological exposure during the puncture phase. In addition, performing balloon inflation and bone cement injection unilaterally reduces the number of operative steps and saves time. This offered shorter prone time and greater tolerance of this procedure in elderly patients, which is similar to the results of previous studies comparing unilateral and bilateral approaches (4, 19). These advantages contribute to enhanced recovery after surgery in patients.

**Table 4:** Intraoperative observation index (*x ± s*).

	Number of penetrations (times)	Bone cement injection volume (ml)	Surgery time (min)
Observation group	37.0 ± 4.7	4.33 ± 0.97	39.8 ± 4.4
Control group	47.1 ± 4.7	4.54 ± 0.76	44.8 ± 5.3
*T*-value	−10.060	−1.116	−4.84
*P*-value	0.000*	0.267	0.000*

*Showed significant differences between the two groups (*P* < 0.05).

The bone cement distribution is an important indicator of vertebral augmentation. Lin (20) showed that the distribution of bone cement can be used as a predictor of the efficacy of unilateral PKP and that uneven cement distribution can lead to vertebral biomechanical imbalance and even vertebral refracture (5). In this study, the bone cement distribution was observed by CT, and the results showed that only 6.8% of the vertebrae in the observation group showed unilateral distribution, which was better than that in previous studies, showing that the superior pedicle notch approach can easily reach the center of the vertebral body, which can effectively reduce the uneven distribution of bone cement and subsequently achieve a similar amount of bone cement injection as the control group. Bone cement leakage is a common complication of vertebral body strengthening, and Klazen (21) observed a leakage rate of up to 72% in PVP using CT in a large-sample multicenter trial. In our center, we also observed bone cement leakage under CT and counted different leakage types separately, and found that the paravertebral leakage rate in the observation group was 25.5%, which was significantly lower than that in the control group (51.2%). This was possibly because the injection target in the observation group was closer to the center of the vertebral body and far from the basin venous foramen and fracture fissure on the lateral wall of the vertebral body, and the pressure gradually decayed when the cement spread to the periphery, thus reducing paravertebral leakage. There were no statistically significant differences in the rates of intradiscal, intervertebral disc, or foraminal leakage between the two groups, indicating that our approach did not increase the risk of these types of leaks. In addition, the results of this study showed that the postoperative VAS scores, OID scores, and vertebral body height improvement rates improved significantly in both groups, and there was no significant difference between the two groups, indicating that short-term pain relief and vertebral body height restoration were comparable between the two approaches.

The superior pedicle notch approach for PKP has some shortcomings. The bony entry point of this approach is located near the intervertebral foramen, which poses a risk of exit-root injury, especially in those with narrow intervertebral foramina due to scoliosis or collapsed intervertebral spaces. In our center, a step-by-step sleeve was used to perform blunt expansion during puncture, which also was used to establish the lumbar endoscopic working channel, and can decrease the risk of injury. This approach was inspired by our experience with percutaneous spinal endoscopy, and we believe it is worth promoting, as it is effective in avoiding nerve root and artery injuries. In addition, a case of asymptomatic leakage of bone cement along the needle tract in the intervertebral foramen region occurred in the observation group. This type of leakage is a potential complication specific to this approach that requires vigilance. It has been reported (22) that good results can be achieved by percutaneous endoscopic removal of pain-causing leaking bone cement.

In conclusion, compared with the bilateral pedicular approach to PKP for lumbar OVCF, unilateral puncture *via* the superior pedicle notch can reach the center of the vertebral body to achieve bilateral cement dispersion, reduce operative time and intraoperative radiation exposure, and decrease the rate of paravertebral cement leakage, while obtaining the same vertebral body height, recovery rate, and clinical efficacy as the bilateral pedicular approach to PKP. In addition, direct access to the vertebral body provides a feasible surgical option for patients with OVCF and poorly developed pedicles and osteoporosis, resulting in poor pedicle visualization and scoliosis.

## Data Availability

The raw data supporting the conclusions of this article will be made available by the authors, without undue reservation.
